# Sex bias in autism spectrum disorder in neurofibromatosis type 1

**DOI:** 10.1186/s11689-016-9159-4

**Published:** 2016-07-26

**Authors:** Shruti Garg, Hein Heuvelman, Susan Huson, Hannah Tobin, Jonathan Green, Northern UK NF1 Research Network, D. Gareth Evans, Elizabeth Howard, Emma Burkitt-Wright, Eileen Hupton, Sonia Patel, Judith Eelloo, Maria Gault, Grace Vasallo, Siobhan West, Vilka Kitching, Karen Tricker, Zahabiyah Bassi, Jamuna Acharya, Wayne Lam

**Affiliations:** 1 Institute of Brain Behaviour and Mental Health, University of Manchester, Oxford Road, Manchester, M13 9PL UK; 2 Child and Adolescent Mental Health Service, Royal Manchester Children’s Hospital, Oxford Road, Manchester, M13 9WL UK; 3 Centre for Biostatistics, Institute for Population Health, University of Manchester, Manchester, UK; 4 Genomic Medicine, Manchester Academic Health Science Centre, Institute of Human Development, The University of Manchester, Central Manchester University Hospitals NHS Trust, St Mary’s Hospital, Oxford Road, Manchester, M13 9WL UK

**Keywords:** Neurofibromatosis type 1, Autism spectrum disorder, Sex bias, Syndromic autism

## Abstract

**Background:**

Despite extensive literature, little is known about the mechanisms underlying sex bias in autism spectrum disorder (ASD). This study investigates the sex differences in ASD associated with neurofibromatosis type 1, a single-gene model of syndromic autism.

**Methods:**

We analysed data from *n* = 194 children aged 4–16 years with neurofibromatosis type 1. Sex differences were evaluated across the Autism Diagnostic Interview-Revised (ADI-R), Autism Diagnostic Observation Schedule (ADOS), verbal IQ, Social Responsiveness Scale (SRS) and Conners questionnaires.

**Results:**

There was 2.68:1 male:female ratio in children meeting ASD criteria on the deep phenotyping measures. On symptom profile, males with neurofibromatosis type 1 (NF1) + ASD were more impaired on reciprocal social interaction and communication domains of the ADI-R but we found no differences on the restricted, repetitive behaviours (RRBs) domain of the ADI-R and no differences on the social on the ADOS. NF1 ASD males and females were comparable on verbal IQ, and the inattention/hyperactivity domains of the Conners questionnaire.

**Conclusions:**

There is a significant male bias in the prevalence of ASD in NF1. The phenotypic profile of NF1 + ASD cases includes greater social communication impairment in males. We discuss the implications of our findings and the rationale for using NF1 as a model for investigating sex bias in idiopathic ASD.

## Background

Sex bias in the prevalence of autism spectrum disorders (ASD) is a widely replicated finding, with a male preponderance in a ratio of 4:1 (male:female) across the whole autism spectrum [1, 2]. Recent studies suggest that a number of methodological issues may influence this ratio, including differences in ascertainment procedures, interaction with intelligence quotient (IQ) and underrepresentation of females in research studies [3]. However, despite such methodological limitations, a relative male preponderance remains a stable observation across all research studies. The male preponderance is not unique to ASD but also seen other neurodevelopmental conditions such as attention deficit hyperactivity disorder (ADHD), dyslexia and developmental language disorders [4]. Study of the mechanisms underlying these sex differences is important as it may help illuminate causal processes within these early onset neurodevelopmental conditions.

In the context of idiopathic ASD, a number of theories have been postulated to explain the apparent sex bias; however, to date, no definitive evidence has emerged to favour any particular one. The most prominent theory is the ‘female protective effect’ (FPE), which suggests that assumed female-specific biological or development factors confer a general protective effect so as to make the effective ASD threshold higher for females [5]. This theory predicts that females with ASD will show higher levels of associated abnormalities compared to males [6]. This prediction is supported by the finding for instance that ASD females show a higher mutational burden, excess deleterious autosomal copy number variations and private single-nucleotide variations [7] as compared to ASD males. A complementary theory postulates male-specific vulnerability or risk factors that lower a threshold to meet ASD criteria. An example would be exposure to foetal testosterone [8] or sex-related genetic factors such as increased ASD risk associated with rare variants genes on the X chromosome such as neurologins 3 and 4 [9], MeCP2 and Fragile X syndrome. At a cognitive trait level, the ‘extreme male brain’ theory proposes that ASD is an extreme manifestation of the normal male brain, with the disorder resulting from exaggerated putative psychological sex differences in empathising and systemizing [10]. A different kind of explanation is that the sex ratio in fact relates to a simple ascertainment bias, resulting from inherent sex biases in the ASD diagnostic criteria and instruments. This explanation maintains that the descriptions of autism and norms established on diagnostic instruments are largely based on research carried out in predominantly male populations [11]; and that symptom exemplars specific to female presentations are not, as a consequence, clearly emphasised in diagnostic instruments [12]. In support of this view, studies investigating differences in behavioural profiles in males and females with ASD suggest decreased levels of restricted, repetitive behaviours in females with no consistent findings in the social communication symptom profile [11, 13]. The largest study to date using the Simons Simplex Collection of *n* = 2418 ASD probands (304 females, 2114 males) found that the females with ASD had greater social communication impairment, lower levels of restricted interests, weaker adaptive skills, greater externalising problems and lower cognitive abilities relative to the males [12]. Similarly, a meta-analysis of 22 studies confirmed lower levels of restricted and repetitive behaviours in females but found no difference in social communication and interaction [14].

A novel route to approach an understanding of the mechanisms underlying sex bias in ASD may be through the study of specific gene disorders causing autism, where the identified causal genetic variant can be assumed to carry a very large proportion of an individual’s autism risk. For example, a study investigating microdeletions of SHANK1 in a four-generation family found that male carriers met the clinical criteria for ASD, whereas female carriers with the same mutation showed evidence of anxiety but not ASD [15]. Similarly, ASD associated with Down’s syndrome shows significantly reduced penetrance in females with the disorder [16]. In a number of these specific gene disorders, considerably more is known about the neurobiology and gene phenotype pathway than in idiopathic autism. Moreover, animal models of syndromic autism can be leveraged to study mechanisms underlying the sex differences.

A strong candidate disorder for this kind of specific gene study is neurofibromatosis type 1 (NF1), a common autosomal dominant single-gene disorder with an estimated birth incidence of 1 in 2700 [17]. Population- and clinical-based studies using gold standard diagnostic ascertainment [18, 19] have demonstrated a prevalence of about 25 % ASD in NF1. Phenotypically, ASD in NF1 shows overall similarity to idiopathic ASD with impairments across the domains of social interaction and restricted repetitive behaviours (RRBs) [20]. IQ is generally in the normal range (mean full scale IQ of 80–90 [21, 22] and presence of IQ <70 in just 4–8 %), and other comorbidity commonly includes attention deficit hyperactivity disorder. Estimate of male:female ratios of ASD in NF1 have ranged between 1.7:1 and 3:1 [18, 19] but this has not, until now, been studied in detail. A recent study using *Nf1* mouse models demonstrated that learning/memory deficits are only observed in male animals [23]. There is considerable inter- and intra-familial variability in the physical phenotypic expression in NF1 [24]. The only sex bias in the physical phenotype reported to date is the elevated risk for optic gliomas in females as compared to males with NF1 [25].

The aim of the present study is to examine sex differences in the behavioural and cognitive phenotype in children with NF1. Based on the findings of previous studies, we investigate whether females with NF1 + ASD show greater symptom severity in the social communication and interaction domain and lower repetitive behaviour symptom levels and cognitive ability when compared to males with NF1 + ASD.

## Methods

### Participants

Data was analysed on children with NF1 and ASD aged 4–18 , drawn from two databases: (1) cohort 1: a genetic registry whole population cohort of children with NF1 aged 4–18 years living in the north of England. A two-stage procedure of Social Responsiveness Scale (SRS) screening followed by gold-standard ASD assessment using Autism Diagnostic Interview-Revised (ADI-R) and Autism Diagnostic Observation Schedule (ADOS) was used. The NICHD-CPEA (National Institute of Child Health and Human Development Collaborative Programs of Excellence in Autism; see procedures for description) criteria were used to identify children with ASD [18]. This study was approved by Greater Manchester South ethics committee (REC reference 11/NW/0838). Data on the SRS was available for *n* = 110 and in-depth phenotyping data from *n* = 46/109 of this database.

(2) Cohort 2: a separate cohort drawn from NF registers at regional genetic centres (Manchester, Leeds, Newcastle, Warrington, Wirral, Alderhey, Sheffield and Edinburgh), as part of an ongoing randomised controlled trial of NF1 + ASD treatment (SimvAstatin in Neurofibromatosis Type 1 Autism (SANTA; EudraCT number: 2012-005742-38, approval granted by Greater Manchester Central Ethics committee, REC reference 13/NW/0111). Here, participant families were recruited via advertisements in NF charities newsletters and social media, as well as clinical referral. Signed informed consent was obtained from all parents and assent from all minor participants. Screen positive children on SRS (*T* > 60) were given in-depth assessment with ADI-R and ADOS. Data on the SRS were available for *n* = 85 and in-depth phenotyping data were available for *n* = 50/85 of this database.

There were significant differences between the NF1 cohorts from the two databases in age (mean age in cohort 1 = 9.87, SD 3.31 and in cohort 2 = 7.45, SD 1.82) and SRS *T* scores (SRS *T* = 63.09, SD 14.10 in cohort 1 and SRS *T* = 78.28, SD 14.52 in cohort 2, *T* = −7.35, *p* < 0.001). Participants were recruited and assessed for the original studies between October 2009 and March 2015.

### Measures

#### SRS 1 and SRS 2

The SRS is a 65-item measure rated on a 4-point Likert scale (1 = never true to 4 = almost always true), which includes five dimensions—social awareness, social cognition, reciprocal social interaction, social motivation and autistic mannerisms. SRS total scores of 76T or higher are in the severe range and are associated with a clinical diagnosis of ASD. It has a sensitivity of 0.85 and specificity of 0.75 for identifying independent consensus expert clinician diagnosis of ASD [26]. Total scores of between 66T and 75T indicate moderately range deficiencies, 60T and 65T indicate mild range deficiencies in reciprocal social behaviour and scores below 59 are considered as being in the normal range. The total *T* scores were computed from the raw scores according to the algorithms available in the SRS-2 manual [27].

#### ADI-R

The ADI-R is a semi-structured, standardised diagnostic interview administered to the caregiver. It consists of 95 items, each of which are coded for current and past behaviour (at age 4–5 years) according to the examiner’s judgement of the presence/absence or the extent of a given behaviour using a scale of 0 (behaviour not present), 1 (behaviour present but not sufficiently severe or frequent to meet criteria for 2), 2 (definite abnormality) or 3 (definite abnormality and marked in severity). The diagnostic algorithm consists of individual behaviour items that have shown good discrimination between groups of children with and without autism. It is based on the most abnormal 4–5-year/ever codes divided into three domains: social interaction, communication and restricted repetitive behaviours [28]. Further, each domain includes four subscales (see Table 2) each, and each subscale includes the number of individual items. A classification of ASD is given when scores in all the three domains exceed the specified cutoffs which is 10 is social interaction domain, 8 in communication and 3 for restricted, repetitive behaviours.

#### ADOS

The ADOS is a standardised interviewer-rated measure for child observation and assessment of skills in communication, social interaction, quality of play and imagination. It is organised into four modules based on the child’s expressive language level. It consists of standardised activities that allow the examiner to observe occurrence of behaviours that have been identified as being important to the diagnosis of ASD. Scores on individual items range from 0 (no evident abnormality) to 3 (marked abnormality). The ADOS-2 diagnostic algorithm was used to score the observations. This algorithm has two domains of ‘social affect’ and ‘restricted repetitive behaviours’ consistent with DSM-5 [29]. An overall total of 7 or higher is classified as meeting criteria for ASD.

#### Conners 3 and Conners Parent Rating Scale-Revised

Conners-3 and CPRS-R short form was used as a measure for ADHD symptoms [30, 31]. It consists of 27 items each rated on a 4-point Likert scale (0 = no true at all to 3 = very much true) in four subscales: oppositional, hyperactivity, cognitive problems and ADHD index.

#### Wechsler Abbreviated Scale of Intelligence (WASI) [32]

The vocabulary and similarity sub-tests of the WASI were used in children over 6 years of age as a measure of intellectual ability.

### Procedure

Medical notes of all patients were reviewed to confirm the diagnosis of NF1 using the National Institutes of Health (NIH) diagnostic criteria [33]. The SRS and Conners questionnaires were completed by the parent or primary caregiver. Detailed phenotyping was carried out using examiner rated ADI-R for parent interviews and ADOS + WASI (verbal sub-scale) for child observation and assessment. The ADI-R assessments were audio-recorded, and the ADOS assessments were video-recorded. All measures were administered by trained researchers and scored during or immediately after administration. Participants were given the research diagnosis of ASD if they met the cutoff criteria on all the three domains of the ADI-R and the overall ASD cutoff criteria on the ADOS.

### Statistical analysis

Data were analysed using SPSS version 17. We compared NF1 males with NF1 females in terms of demographics and clinical characteristics using two-sample *t* tests for continuous data and *χ*^2^ tests for categorical data (Table 1). In the subset who had received in-depth clinical assessment, we compared males and females in terms of means and standard deviations on ADI-R subscales, ADOS, Conners, SRS, and verbal IQ using two-sample *t* tests (Table 2). Finally, the ASD males were compared with ASD females on ADI-R subscales, ADOS, Conners, SRS and verbal IQ using the two-sample *t* test; *p* values and the standardised mean differences are reported (Table 3). The critical *p* values for significance were Bonferroni adjusted.

**Table 1:** Demographics and SRS treatment scales of the total sample of *n* = 194 children with NF1

	Males (*n* = 103)	Females (*n* = 91)	Test statistic	*p* value*
Age, mean (SD)	8.6 (2.9)	9.2 (3.1)	*T* = −1.35	0.193
Familial inheritance, *n* ^a^	48	45	*χ* ^2^ = 0.72	0.698
Educational statement, *n* ^b^	17	18	*χ* ^2^ = 0.11	0.744
SRS (raw scores), mean (SD)				
Social Communication and Interaction (SCI) domain	71.57 (32.91)	55.77 (29.79)	*T* = 3.49	0.001
Social awareness	11.60 (4.81)	9.30 (4.36)	*T* = 3.48	0.001
Social cognition	17.02 (8.98)	12.98 (7.98)	*T* = 3.29	0.001
Social communication	29.65 (15.06)	22.43 (13.14)	*T* = 3.53	0.001
Social motivation	13.30 (6.94)	11.07 (7.05)	*T* = 2.22	0.001
Restrictive and repetitive behaviours domain	17.93 (8.67)	13.56 (8.56)	*T* = 3.52	0.001
SRS total raw score	89.50 (40.74)	69.33 (37.48)	*T* = 3.57	<0.001

*Significant if *p* < 0.005

^a^Disease inheritance data available for *n* = 88 males and *n* = 80 females

^b^Educational statement data available for *n* = 77 males and *n* = 74 females

**Table 2:** Characteristics of NF1 males versus NF1 females on ADI-R subscales, Conners, SRS and verbal IQ

	Males (*n* = 54)	Females (*n* = 42)	*t* test	*P* value*
Mean (SD)				
ADI-R: reciprocal social interaction (A)				
Failure to use non-verbal behaviours to regulate social interaction (A1)	3.61 (1.74)	1.74 (1.45)	4.98	<0.001
Failure to develop peer relationships (A2)	5.19 (2.53)	3.57 (2.56)	3.08	0.003
Lack of shared enjoyment (A3)	3.30 (1.75)	1.71 (1.67)	4.47	<0.001
Lack of socio-emotional reciprocity (A4)	4.63 (2.26)	2.76 (2.09)	4.15	<0.001
ADI-R: communication (B)				
Lack/delay in spoken language and failure to compensate through gestures (B1)	2.85 (2.56)	1.57 (2.11)	2.62	0.01
Lack of varied make-believe play (B4)	3.96 (2.06)	2.06 (2.28)	2.92	0.004
Relative failure to initiate/sustain conversational interchange (B2)	2.83 (1.40)	1.45 (1.23)	5.05	<0.001
Stereotyped, repetitive or idiosyncratic speech (B3)	3.33 (1.49)	2.10 (1.96)	3.51	0.001
ADI-R: restricted, repetitive and stereotyped patterns of behaviour (C)				
Encompassing preoccupation or circumscribed pattern of interest (C1)	1.57 (1.34)	0.66 (0.96)	3.70	<0.001
Apparently compulsive adherence to non-functional routines or rituals (C2)	1.44 (1.29)	1.19 (1.42)	0.91	0.364
Stereotyped and repetitive motor mannerisms (C3)	0.57 (0.82)	0.52 (0.80)	0.30	0.764
Preoccupation with parts of objects or non-functional elements of material (C4)	1.20 (0.88)	0.74 (0.73)	2.77	0.007
ADOS-SA total	8.11 (4.42)	4.52 (4.03)	4.10	<0.001
ADOS-RRB total	1.41 (1.54)	0.69 (1.33)	2.40	0.018
ADOS overall total (SA + RRB)	9.48 (5.28)	5.50 (4.46)	3.92	<0.001
Conners-inattention^a^	67.97 (13.64)	68.03 (14.41)	−0.02	0.981
Conners-hyperactivity^a^	67.19 (15.53)	66.82 (16.64)	0.14	0.890
SRS *T* scores	72.17 (16.09)	67.00 (15.81)	2.25	0.025
Verbal IQ	89.98 (14.19)	95.38 (16.36)	−1.67	0.099

^a^Conners data available for *n* = 78 males and *n* = 67 females. *Significant if *p* < 0.002

**Table 3:** Characteristics of NF1 ASD males versus NF1 ASD females on ADI-R subscales, Conners, SRS and verbal IQ

	ASD males, (*n* = 31)	ASD females (*n* = 9)	*t* test	*P* value*	Standardised mean difference (Cohen’s *d*)	
					Est.	(95 % CI)
Mean (SD)						
ADI-R: reciprocal social interaction (A)						
Failure to use non-verbal behaviours to regulate social interaction (A1)	4.55 (1.54)	2.44 (1.01)	3.83	<0.001	1.451	(0.640, 2.262)
Failure to develop peer relationships (A2)	6.63 (1.56)	6.0 (1.12)	1.13	0.267	0.428	(−0.323, 1.179)
Lack of shared enjoyment (A3)	4.16 (1.32)	2.67 (1.87)	2.72	0.010	1.029	(0.252, 1.806)
Lack of socio-emotional reciprocity (A4)	5.74 (1.75)	4.33 (2.40)	1.94	0.058	0.739	(−0.021, 1.500)
ADI-R: communication (B)						
Lack/delay in spoken language and failure to compensate through gestures (B1)	4.32 (2.29)	2.33 (2.29)	2.29	0.027	0.870	(0.102, 1.637)
Lack of varied make-believe play (B4)	5.13 (1.28)	4.11 (1.17)	2.13	0.039	0.808	(0.044, 1.572)
Relative failure to initiate/sustain conversational interchange (B2)	3.58 (0.72)	2.33 (1.50)	3.51	0.001	1.327	(0.528, 2.127)
Stereotyped, repetitive or idiosyncratic speech (B3)	3.68 (1.56)	3.67 (2.45)	0.02	0.987	0.006	(−0.736, 0.748)
ADI-R: restricted, repetitive and stereotyped patterns of behaviour (C)						
Encompassing preoccupation or circumscribed pattern of interest (C1)	2.23 (1.33)	1.38 (1.51)	1.57	0.125	0.622	(−0.168, 1.412)
Apparently compulsive adherence to non-functional routines or rituals (C2)	1.87 (1.15)	2.67 (1.23)	−1.81	0.079	−0.684	(−1.441, 0.074)
Stereotyped and repetitive motor mannerisms (C3)	0.71 (0.90)	1.11 (1.05)	−1.13	0.264	−0.429	(−1.177, 0.319)
Preoccupation with parts of objects or non-functional elements of material (C4)	1.61 (0.76)	1.11 (0.78)	1.73	0.091	0.656	(−0.101, 1.412)
ADOS-SA total	10.45 (3.44)	8.33 (2.23)	1.73	0.091	0.656	(−0.100, 1.413)
ADOS-RRB total	2.16 (1.59)	1.67 (2.18)	0.75	0.456	0.285	(−0.460, 1.030)
ADOS Overall total (SA + RRB)	12.61 (3.96)	10.0 (3.24)	1.81	0.079	0.683	(−0.074, 1.441)
Conners-inattention	76.03 (11.17)	80.0 (16.04)	−0.78	0.439	−0.327	(−1.151, 0.496)
Conners-hyperactivity	75.19 (12.26)	77.0 (22.20)	−0.30	0.766	−0.125	(−0.946, 0.695)
SRS total *T* scores	84.35 (10.12)	85.33 (9.49)	−0.259	0.797	−0.098	(−0.840, 0.644)
Verbal IQ	88.50 (12.95)	90.38 (10.96)	−0.370	0.714	−0.149	(−0.943, 0.644)

*Significant if *p* < 0.002

## Results

### Demographic and clinical characteristics of the sample

SRS data were available for *n* = 194 children with NF1, of whom *n* = 101 (53.09 %) were male. The mean age of the sample was 8.8 years (SD 2.0, range 4.0–16.5). The pattern of inheritance was familial in *n* = 93, de novo in *n* = 75, and unknown in *n* = 26 cases. There were no sex differences in age, familial pattern of inheritance and in having a statement of special educational needs (see Table 1).

**Fig. 1:**
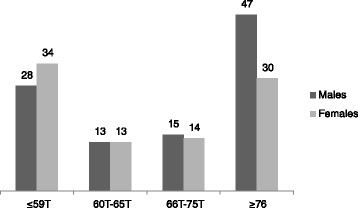
SRS total *T* scores in males and females in the whole sample *n* = 194

On the SRS questionnaire, males had significantly higher raw scores than females on all of sub-domains. The proportion of males with *T* scores ≤59, 60 to 65, 66 to 75 and ≥76 were 27.2, 12.6, 14.6 and 45.6 %, respectively. The proportion of females with *T* scores ≤59, 60 to 65, 66 to 75 and ≥76 were 37.4, 14.3, 15.4 and 33.0 %, respectively (See Fig. 1).

### Sex comparison on the detailed phenotyping measures

In-depth phenotyping data were available for *n* = 54 males and *n* = 42 females (total *n* = 96, 49.5 % of the total sample). There were no differences between groups with and without in-depth phenotyping data in terms of age (8.6 (2.6) versus 9.1 (3.4), *t* = −1.12, *p* = 0.223) or sex (*χ*^2^ = 0.18, *p* = 0.667) but the SRS *T* scores were higher in the group with the in-depth phenotyping data (77.2 (13.3) versus 62.2 (15.3), *t* = 7.16, *p* < 0.000) (Table 2).

On the ADI-R, significant sex differences were observed on (i) three sub-scales in the reciprocal social interaction domain failure to use non-verbal behaviours to regulate social interaction, lack of shared enjoyment and lack of socio-emotional reciprocity, (ii) two sub-scales in communication domain failure to sustain conversational interchange and stereotype speech and (iii) preoccupation/circumscribed patterns of interest sub-scale of the restricted, repetitive behaviours domain. Males were significantly more impaired than females on the ADOS Social Affect domain and had significantly higher ADOS total scores. There were no statistically significant sex differences on verbal IQ and Conners inattention/hyperactivity sub-scales.

### Sex differences on the sample that met the ASD cutoff criteria on the ADI-R and ADOS

Of the children with in-depth phenotyping data, the ASD cutoff criteria was met by 57.4 % (31/54) males and 21.4 % females (9/42) (*χ*^2^ = 12.58, *p* < 0.001). The mean age of ASD males was 7.96 (SD 1.7) and females 8.61 (SD 3.27) (*t* = −0.81, *p* = 0.422).

Table 3 shows the detailed scores on the ADI-R subscales, Connors, SRS and verbal IQ amongst males and females with ASD. The only significant differences were observed on two subscales of the ADI-R failure to use non-verbal behaviours to regulate social interaction and failure to initiate conversational interchange on the social communication domain. No sex differences were observed on the restricted and repetitive domain of the ADI-R. Similarly, there were no sex differences on the ADOS social affect, repetitive behaviour or the ADOS total scores. There were no significant differences on the Conners inattention/ hyperactivity domains, verbal IQ or the SRS total scores. The standardised mean differences between the groups is also reported which suggest large effect sizes for the reciprocal social interaction and communication domains of the ADI-R (other than failure to develop peer relationships A2 and stereotyped/idiosyncratic speech B3 sub-scales).

## Discussion

To our knowledge, this is the first study investigating sex differences in ASD using a syndromic model of autism. We found a significant sex bias in ASD prevalence at a ratio of 2.68:1 males:females. This latter finding is similar to the rates reported in the idiopathic ASD literature in children with average or below average IQ and is consistent with findings reported by Plasschaert et al. who found similar rates of sex bias in a clinic-ascertained NF1 + ASD sample (*n* = 27) [19]. The groups in our study were well matched on co-morbid ADHD symptomatology and verbal IQ. In the context of other specific gene syndromic autism, our findings of a male bias in NF1-ASD are similar to results in SHANK1 [15] and Down’s syndrome [16]; however, this sex disparity is not seen in genetic disorders associated with high levels of intellectual impairment, such as tuberous sclerosis [34] and Cornelia de Lange syndrome [35].

Regarding the phenotypic profile, we found that NF1 males showed overall greater social communication impairments on the SRS, ADI-R and ADOS (Tables 1 and 2). However, no statistically significant differences were observed between males and females on Conners inattention/hyperactivity domains and on verbal IQ. Given the small samples sizes of the NF1 + ASD sub-group (Table 3), we report the standardised mean differences between ASD males and females. ASD males are more impaired in the social interaction and communication domains. There are no significant sex differences in the NF1 + ASD sample on the ADOS total and SRS *T* scores, nor in ADHD symptomatology or verbal IQ. These findings are therefore in contrast to those reported in the idiopathic ASD literature, in which ASD females have been reported to have more social communication impairment, lower levels of repetitive behaviours, greater externalising problems and lower cognitive abilities as compared to ASD males [12]. However, these results are limited by small sample sizes of NF1 females (*n* = 9, Table 3) and need to be replicated in larger samples.

How might the results of sex bias in NF1 ASD be explained? The NF1 gene encodes for protein neurofibromin, which in turn regulates different downstream signalling effectors such as cAMP, dopamine and Ras/mammalian target of rapamycin (mTOR). Using *Nf1* genetically engineered animal model, a recent study has demonstrated that increased glioma risk in females with Nf1 is linked to a modifier gene and sexually dimorphic cAMP signalling [25]. However, the molecular mechanisms for sex-specific modifiers in NF1 ASD are poorly understood. One plausible hypothesis could be the role of sex hormones in mediating differential sensitivity to downstream effectors. Another hypothesis may be that sex interacts with germline NF1 mutation through epigenetic mechanisms to produce changes in gene expression [23]. Altogether, the data is compatible with the female protective effect, although the exact mechanisms for this are unclear. Simple measurement ascertainment bias is not supported by our data, since, between sex-specific groups that are otherwise balanced for IQ and co-morbidity, the male bias is apparent on the parent-rated dimensional SRS measure as well as researcher rated in-depth phenotyping measures. Further, prospective studies of sex-specific developmental trajectories in NF1 could help clarify how the gene environment interaction may contribute to the emergence of sex differences in NF1 ASD. It would also be useful to better understand the sex differences in the ASD phenotype in other models of syndromic autism.

These findings should be interpreted in light of the study’s limitations. Our study samples were drawn from two separate cohorts with significant differences in age and SRS *T* scores; however, we think that it is unlikely that this influences out interpretations of the results. The sample from cohort 2 was part of a RCT using Simvastatin treatment in children with NF1 ASD; participants were actively recruited in the RCT on the basis of ASD symptomatology on the SRS. There is no evidence however that the different origin of the included cohort influenced the analysis findings. Secondly, we acknowledge the relatively small sample size of the NF1 + ASD females, thus limiting the conclusions that can be drawn from the data presented in Table 3. Nonetheless, this is a novel approach to the investigation of sex differences in ASD in a single-gene syndromic model, which gives strength to our interpretation.

## Conclusions

The present study suggests a significant male bias in the incidence of NF1 + ASD. The phenotypic profile of NF1 + ASD cases includes greater social communication impairment in males but comparable levels of RRBs, ADHD symptomatology and cognitive abilities. Our data support the possibility of sex-specific modifiers in NF1 which confer a female protective effect to ASD in neurodevelopment and suggests that NF1 is a strong candidate model for investigation of sex differences in ASD.

## Abbreviations

ASD, autism spectrum disorder; ADI-R, Autism Diagnostic Interview Revised; ADOS, Autism Diagnostic Observation Schedule; ADHD, attention deficit hyperactivity disorder; CPEA, Collaborative Programme of Excellence in Autism; DSM, Diagnostic and Statistical Manual of mental disorders; NF1, neurofibromatosis type 1; NICHD, National Institute of Child Health and Human Development; RRB, restricted repetitive behaviours; SRS, Social Responsiveness Scale
